# “For aging not passively endured, but actively experienced:” on *Symposium on Gerontology* from 1958

**DOI:** 10.3325/cmj.2020.61.167

**Published:** 2020-04

**Authors:** Stella Fatović-Ferenčić, Martin Kuhar

**Affiliations:** The Division for the History of Medical Sciences, Department of the History and Philosophy of Science, Croatian Academy of Sciences and Arts, Zagreb, Croatia

## Abstract

The subjects of gerontology and geriatrics did not arouse stronger interest among Croatian scholars until the second half of the twentieth century. From 1952 to 1957, a number of Croatian medical experts gave lectures on gerontology at the Yugoslav Academy of Sciences and Arts. Based on these lectures, in 1958 the Academy published the first book on gerontology in Croatia under the title *Symposium on Gerontology*. Its editor was Franjo Kogoj, a dermatovenereologist and a Fellow of the Academy (1894-1983). In this article, we focused on the contents of *Symposium*, namely, on the discussions about geriatric terminology, theories of aging, epistemological issues in gerontology, as well as clinical experiences with older patients. We argue that *Symposium* marks the beginning of a synthetic and interdisciplinary approach to gerontology in Croatia.

Gerontology became a topic of interest among Croatian researchers only in the second half of the twentieth century. From 1952 to 1957, prominent Croatian medical experts held several lectures about gerontology at the Department of Medical Sciences of the Yugoslav Academy of Sciences and Arts. On the basis of these lectures, in 1958 the Academy published the first book on gerontology in Croatia – *Simposion o gerontologiji* [*Symposium on Gerontology*]. The book was edited by Franjo Kogoj (1894-1983), the Secretary of the Department of Medical Sciences and the Head of the Clinic for Skin and Venereal Diseases in Zagreb (1).

Since many historical overviews of gerontology have been published both internationally and in Croatia (2-4), we focused here on the contents of *Symposium*, which so far has not stirred the scholarly interest. *Symposium* encompasses discussions on the terminology, theories of aging, epistemological issues in gerontology, as well as clinical experiences with older patients. As such, this publication represents the beginnings of an interdisciplinary approach to the topic of gerontology in Croatia.

## The traces of gerontology in Croatia in the first half of the 20th century

The intensive development of chemistry and biology in the first half of the 20th century led to the development of many theories of aging. As one of the most prominent representatives of the bacteriological era, Russian biologist and Nobel Prize winner Ilya Mechnikov, who coined the term gerontology, postulated that aging resulted from a slow and chronic poisoning by the toxins produced by the bacteria in the large intestine. In his work The Prolongation of Life: Optimistic Studies, Mechnikov described the potential life-lengthening properties of lactic acid bacteria and recommended regular consummation of yoghurt to halt the process of aging (5). On the other hand, the father of geriatrics was Leo Ignaz Nascher, who proposed that name “to cover the same field in old age that is covered by the term pediatrics in childhood, to emphasize the necessity of considering senility and its disease apart from maturity and to assign it a separate place in medicine” (6).

The phenomenon of aging stimulated not only theoretical reflections but also a series of rejuvenation experiments. In the first half of the twentieth century, Paul Niehans suggested fetal injections, a procedure apparently tried on Winston Churchill and Pope Pius XII (7). In Romania in 1949, Ana Aslan invented Gerovital H3, a procaine-based medicine that allegedly delayed the aging process. By selling this remedy, she made enough money to establish the Institute of Geriatrics in Bucharest in 1952 and became well respected in gerontological circles (8). The International Association of Gerontology was founded in Liege in 1950, when the first congress on gerontology was held. Next year, the congress was held in St Louis, and in 1954 in London.

Croatia significantly lagged behind these developments. While the international bibliography of geriatric literature at that time contained around 18 000 references, the papers on this topic in Croatia were almost nonexistent. In 1907, Croatian pioneer of sexology and venereology Fran Gundrum published a macrobiotic discussion on the prolongation of life and hygienic measures for the prevention of aging. In 1918, county physician within the governmental health department Artur Lang described a case of senile dementia in an 87-year-old woman (9), while a similar paper by Zvonimir Sušić was published in 1945 under the title Mental Disturbances in Old Age and Civil Competence (10). Both articles reflect the need to establish whether senile dementia existed in a patient and thus declare his or her legal incompetence as part of forensic evaluations. It was Croatian public health pioneer Andrija Štampar who first anticipated public health issues regarding the aging population. In his book *Higijena i socijalna medicina* [*Hygiene and Social Medicine*] from 1940, Štampar analyzed and compared vital statistics from various European countries, and showed how the decrease in birth rates and the prolongation of life would change “economic and social structure” and necessitate the introduction of “new social and health measures” (11).

## Discussions on aging within the Academy’s Department of Medical Sciences

In 1947, Andrija Štampar was elected full member of the Academy, and already in the next year became its President (1948-1958). During his presidency, he remained focused on public health and successfully collaborated with the Department of Medical Sciences, which was led by Franjo Kogoj. During Kogoj’s mandate (1950-1958), two important works were published: *Symposium on Allergy* in 1954 and *Symposium on Gerontology* in 1958, both edited by Kogoj.

Between 1952 and 1957, members of the Section for Medical Sciences of the Academy’s Department of Medical Sciences gave lectures on various topics in gerontology. The first lecture was held by Pavle Sokolić, the founder of the Institute for Pathophysiology at the School of Medicine in Zagreb, on June 11, 1952 under the name Physiological and Pathophysiological Views on the Problem of Aging, and the final by neuropsychiatrist Radoslav Lopašić on July 10, 1957 under the name Neurological and Psychiatric Disturbances in Involution. The lectures formed the basis of *Symposium on Gerontology*, which contained 14 chapters. Apart from the Foreword, Franjo Kogoj penned the introductory chapter, titled Some General Remarks Concerning Gerontology, as well as the final chapter dealing with geriatric dermatology. The chapter Reflections on Basic Problems and the Historical Development of Gerontology and Geriatrics was authored by medical historian Mirko Dražen Grmek, the chapter Aging of the Yugoslav Population by Branko Kesić, the chapter Progressive Processes in Old Age by Zvonimir Kopač, Problems of Old Age and Aging in Internal Medicine by Arpad Hahn, *Morbus senilis cordis* by Dinko Sučić, Tuberculosis and Old Age by Stanko Ibler, Surgery in Old Age by Dimitrije Juzbašić, Aging of the Stato-acoustic Apparatus in Humans by Branimir Gušić, The Role of Lymphatic System in Aging by Srećko Podvinec, Gynecologic Geriatrics by Franjo Durst, Aging of the Human Skeleton with Special Emphasis on the Bones of the Skull and Jaws by Ivo Čupar, and Neurological and Psychiatric Disturbances in Involution by Radoslav Lopašić. Pavle Sokolić did not manage to finish his text on time, so it was not included in the final publication. Given the breadth of its topics, *Symposium on Gerontology* can be seen as a reference book on the terminology, theories of aging, as well as definitions and timeframe of old age. Despite the fact that all the contributors to *Symposium* were physicians, Kogoj encouraged them to approach the topic of gerontology synthetically rather than from a strictly specialized perspective.

Kogoj’s introductory chapter reveals a scholar well-versed in the international debates in gerontology and aware of many blanks still waiting to be filled on this topic in Croatia. Other authors were aware of this as well; for example, pathologist Zvonimir Kopač claimed that the “current state of the science on old age is still in the phase of data collection and the formulation of more or less successful hypotheses” (12). *Symposium* also contained discussions on various theories of aging, brought in a most detailed fashion by the editor, Franjo Kogoj. He supported the notion that one of the main reasons of premature aging was “life stress,” due to the “incessant attacks on vegetative and sensible nervous system” (13). At the same time, he rejected the idea that aging was a linear process equally affecting all tissues and organs, and accepted that aging was individually, even “constitutionally,” determined. Most importantly, he did not think that one should simply succumb to the fate of aging, and advocated for an old age that would not be “passively endured, but actively experienced” (13). Such theoretical framework led to some practical recommendations, especially regarding the need to maintain physical and mental shape in old age: “General and individual prevention of illness and its careful treatment is the first and main condition for the coordinated and harmonic development of senescence and senium” (13). To add to his elaborations, Kogoj included a number of illustrations by famous artists such as Anthony van Dyck and Leonardo da Vinci that to him represented “good” and “bad” aging.

Most contributors to *Symposium* reflected on the definitions of gerontology, old age, and aging. While Grmek commented on the widely-accepted definitions of gerontology and geriatrics, phthisiologist Stanko Ibler proposed the use of M. Bürger’s term “biomorphosis,” emphasizing that the processes responsible for aging began with the conception (14). He also remarked on the definition of old age, claiming that it should incorporate its inherent “historical character” (14). Namely, Ibler held that medicine alone could not ascertain when old age begins, since the answer to that question depended upon “certain societal expectations” as well (14). An even broader view of old age and aging was given by Kopač, who wrote that “the life of higher species is not only depletion, rather, it is giving birth and dying at the same time, wasting and creating, aging and rejuvenating of the same individual, of his different parts and his myriad cells, tissues and juices” (12).

Even though he accepted the notion that certain tissues and organs could show growth and regeneration, internist Arpad Hahn still maintained that the main symptom of aging was atrophy. He made a detailed physical description of external bodily changes in old age and an overview of morphological and functional alterations of all systems, tissues, and organs of the human body. Apart from that, Hahn observed that an old body reacts in an “incomplete” fashion when ill, and that many symptoms are “abortive and frustrated” (15). Combined with numerous bodily changes in old age, this fact could lead, Hahn claimed, to wrong diagnoses. While a clinician dealing with a younger person should employ a synthetic approach, linking various symptoms and signs to reach a diagnosis, Hahn recommended an “analytic way of thinking,” with older patients that had to accomplish two things: first, separate general changes in old age from the pathological changes, and second, take into consideration that the latter could appear stunted and thus mask the seriousness of the condition (15).

*Symposium* also contained the descriptions of first clinical experiences with elderly patients from two Croatian hospitals. Dinko Sučić, cardiologist and Head of the Internal Medicine Ward at Dr Mladen Stojanović Clinic (today’s Clinical Hospital Sestre Milosrdnice), noted that myocarditis only accounted for 3% of the hospitalized patients, while “myodegeneration” was present in 53.7%. The average age for the diagnosis of myodegeneration was 63, and men were twice as likely to be diagnosed as women. Claiming that there did not exist a single accepted nomenclature of heart disease in old age, and that the “pathoanatomical substrate” was frequently either incomplete or nonexistent, Sučić suggested the clinical diagnosis *morbus senilis cordis* for all changes in the hearts of older patients (16). Sučić was thus the first cardiologist in Croatia to approach the rising public health issue of heart disease in the context of geriatric population. Similarly, surgeon Dimitrije Juzbašić, who in 1953 came to the Clinic of Surgery of the School of Medicine in Zagreb and introduced new operative techniques, developed experimental work, and created conditions for scientific research, published his clinical results in *Symposium*. Juzbašić stated that in 1955, 126 patients above the age of 70 were hospitalized in his Clinic. Of those, 63 were operated on, with the mortality of 6.3%, while the operations were characterized as moderately difficult or difficult. Concluding his text, Juzbašić emphasized the need to carefully plan the operation among elderly patients and take into consideration how it would affect the quality of life (17).

Two authors of *Symposium* discussed sex differences in the onset, speed, and intensity of aging: gynecologist and obstetrician Franjo Durst and neuropsychiatrist Radoslav Lopašić. Durst remarked that the climacterium signaled the beginning of old age in women, and described it as a typical female phenomenon that separated women from men both mentally and physically. Also, he linked the climacterium and senium with the higher probability for breast cancer (18). Similarly, in his analysis of mental changes in old age, Lopašić stated that involution begins a little earlier in women due to the climacteric changes, which are also characterized by specific mental issues. Noting the rising number of old people and, consequently, those “senile and abnormally senile,” Lopašić emphasized new tasks awaiting the health service. Thus, he advocated the establishment of geriatric wards in hospitals and mental institutions, building of new nursing homes, organizing community-health nursing, and mental-hygienic and psychotherapeutic work with the elderly (19).

In the context of a new pathocenosis in the second half of the twentieth century, in which chronic and degenerative diseases rose in both absolute and relative numbers as the population aged, new and different public health issues were prioritized. Within *Symposium*, an important place belongs to the analysis of vital statistics of Yugoslavia made by specialist of hygiene and social and occupational medicine Branko Kesić. In his article on the aging of Yugoslav population, Kesić noted that the age structure significantly differed between the 1931 and 1951 censuses, with the main changes being the higher life expectancy and a lower number of children. That Kesić derived his approach from Štampar’s social medicine was clear when he stated that the main culprits of the population aging were industrialization and rural to urban migration. As a consequence of these processes, fertility was declining, while life conditions and health care improved, which all affected the prolongation of life. The aging population, according to Kesić, represented a social and economic problem that would need to be tackled with the prevention of geriatric pathology, degenerative diseases, and disability. Kesić also claimed that medicine had to solve these problems not only because of humanitarian reasons but also because of social and economic reasons. Healthcare and social insurance had to anticipate all measures that needed to be applied, such as the building of various institutions that could assuage the disabilities that accompany aging (20). Kesić’s article thus added to Štampar’s thoughts on the population structure from 1940, with a clear formulation of public health concerns that the changing vital statistics brought.

Generally speaking, the contents of *Symposium* reflected the views of prominent representatives of medical profession in Croatia, some of whom were members of the Academy. Most of the chapters were written by clinicians, who presented detailed clinical symptomatology of old age through their specialties, thus advocating the introduction of subspecialties. For example, in his chapter on skin changes and diseases in old age, Franjo Kogoj concluded that “there existed a gerontologic and a geriatric dermatology” (21). On the other hand, physicians were still rooted in a holistic approach to pathology, clearly seen in their many references to the geriatric terminology and nosology, theories of aging, and social problems derived from an aging population. Taking all this into consideration, physicians understood that they only scratched the surface of the subject of gerontology. One of them was Ivo Čupar, the founder of orthognathic surgery in Croatia, who wrote in *Symposium* that “although we can find some answers, in the end we have to be content with the fact that the life and death of human tissues are still a mystery to us” (22).

## Discussion

The most comprehensive overview of the history and development of gerontology in the Croatian language was published in *Symposium*. In that text, historian of medicine Mirko Dražen Grmek gave a detailed chronology of gerontology and geriatrics both internationally and in Croatia (2). The next medico-historical study on the topic of gerontology was written by Biserka Belicza and dealt with the authors writing about old age, aging, and dying until the 17th century (3). The extreme scarcity of gerontological literature in the first half of the twentieth century in Croatia (23) in part stemmed from the poverty and wartime destructions, as well as the slower aging of the Yugoslav population in comparison with more developed industrialized nations (20). A particularly difficult issue for the Croatian public health pioneers was the high infant and child mortality, which shifted the health care priorities toward the prevention of premature death. Unsurprisingly, problems of the elderly were mostly left aside, at least until the middle of the 20th century, when our public health experts Andrija Štampar and Branko Kesić first detected significant demographic changes in the Yugoslav population (11,20). Therefore, a need arose for a detailed and comprehensive discussion on gerontology from a variety of viewpoints, which also included clinical experts. Thus, the authors of *Symposium*, by referencing many foreign authors who already extensively debated on the topic of aging and its problems, argued for the need to establish a specific approach within every medical specialty toward the elderly.

It can be safely assumed that *Symposium* stimulated new debates within gerontology in Croatia in the second half of the twentieth century. This assumption is supported by the fact that some of the authors of *Symposium* contributed to the entry Gerontology and Geriatrics in *Medical Encyclopedia*, thus opening up space for such novel issues within standard national medical literature. In that text, Grmek analyzed the definitions of gerontology and geriatrics and gave a brief historical overview of major breakthroughs in the field, Kopač wrote about pathological processes in old age, Lopašić about the neuropsychiatric diseases among the elderly, and Juzbašić about surgery in older patients (24). The only new author contributing to this entry was Miloš Škarica, the Chief of the Internal Medicine Ward in Zadar General Hospital, who penned the discussion on the diseases affecting internal organs of the elderly, as well as the text on caring for older people. Škarica also added to the foreign discussions about rejuvenation with his 1959 book *Kako ćemo produžiti život* [*How can we prolong life*] (25). In 1969, the Croatian Medical Association organized a three-day congress on geriatrics and published a book of the conference proceedings. One of the main aims of this congress was to stimulate a broader societal action in the field of health care and social protection of the elderly, especially in Zagreb. One of the links between this congress and *Symposium* was Branimir Gušić, whose presence affirmed the continuity and importance of this topic in the decade after the publication of *Symposium* (26). Despite these developments, in the chapter on geriatrics in the book *Medical Specialties* from 1981, Miloš Škarica states: “Great many books and thousands of journal articles about gerontology exist in the foreign literature. On our territories, unfortunately, precious little has been done until the end of the Second World War (a lot of educated men still do not know the meaning of geriatrics and gerontology)” (27).

## Conclusion

Although the historiography of gerontology rarely mentions *Symposium on Gerontology*, this is a publication that definitely marks the beginning of a comprehensive treatment of this important topic in Croatia. *Symposium* correctly anticipated new health care priorities and views on gerontology and geriatrics in the region. With its interdisciplinary approach that encompassed the definitions of gerontology and geriatrics, historiography, theories of aging, physiology, pathology, and clinical as well as public health implications of aging, this publication represents the pioneering work on this topic. At the same time, the authors of *Symposium* influenced both the creation and the future development of gerontology in Croatia.

## References

[1] Kogoj F, editor. Simposion o gerontologiji. Zagreb: JAZU; 1958.

[2] Grmek MD. Razmatranja o osnovnim problemima i povijesnom razvoju gerontologije i gerijatrije. In: Kogoj F, editor. Simposion o gerontologiji. Zagreb: JAZU; 1958. p. 21-102.

[3] Belicza B (1997). Croatian gerontology from the ancient times to the seventeenth century.. Croat Med J.

[4] Belicza B, Vondraček-Mesar J (1997). Ageing and dying in Croatian rural community at the end of the 19th and beginning of the 20th century.. Croat Med J.

[5] Podolsky SH (2012). Metchnikoff and the microbiome.. Lancet.

[6] Nascher JL (1909). Geriatrics.. NY Med J..

[7] Morley JE (2004). A brief history of geriatrics.. J Gerontol.

[8] Dumitrascu DL, Shampo MA, Kyle RA (1998). Ana Aslan – founder of the First Institute of Geriatrics.. Mayo Clin Proc.

[9] Lang A (1918). Dementia senilis simplex.. Lijec Vjesn.

[10] Sušić Z (1945). Psihičke smetnje staračkog doba i građanska sposobnost.. Lijec Vjesn.

[11] Štampar A. Higijena i socijalna medicina. Zagreb: Tisak Zaklade tiskare Narodnih novina; 1940.

[12] Kopač Z. Progresivni procesi u starosti. In: Kogoj F, editor. Simposion o gerontologiji. Zagreb: JAZU; 1958. p. 129-40.

[13] Kogoj F. Nekoliko općih napomena o gerontologiji. In: Kogoj F, editor. Simposion o gerontologiji. Zagreb: JAZU; 1958. p. 9-20.

[14] Ibler S. Tuberkuloza i starost. In: Kogoj F, editor. Simposion o gerontologiji. Zagreb: JAZU; 1958. p. 187-202.

[15] Hahn A. Problemi starenja i starosti u internoj medicini. In: Kogoj F, editor. Simposion o gerontologiji. Zagreb: JAZU; 1958. p. 141-72.

[16] Sučić D. Morbus senilis cordis. In: Kogoj F, editor. Simposion o gerontologiji. Zagreb: JAZU; 1958. p. 173-86.

[17] Juzbašić D. Kirurgija u starosti. In: Kogoj F, editor. Simposion o gerontologiji. Zagreb: JAZU; 1958. p. 203-17.

[18] Durst F. Ginekološka gerijatrija. In: Kogoj F, editor. Simposion o gerontologiji. Zagreb: JAZU; 1958. p. 249-89.

[19] Lopašić R. Neurološke i psihijatrijske poremetnje u involuciji. In: Kogoj F, editor. Simposion o gerontologiji. Zagreb: JAZU; 1958. p. 305-56.

[20] Kesić B. Starenje jugoslavenskih naroda. In: Kogoj F, editor. Simposion o gerontologiji. Zagreb: JAZU; 1958. p. 103-28.

[21] Kogoj F. Cutis senilis. In: Kogoj F, editor. Simposion o gerontologiji. Zagreb: JAZU; 1958. p. 357-73.

[22] Čupar I. Pojave starenja na ljudskom kosturu s osobitim obzirom na kosti lubanje i čeljusti. In: Kogoj F, editor. Simposion o gerontologiji. Zagreb: JAZU; 1958. p. 291-303.

[23] Grmek MD. Hrvatska medicinska bibliografija. Zagreb: JAZU; 1970.

[24] Grmek MD, Kopač Z, Škarica M, Lopašić R, Juzbašić D. Gerontologija i gerijatrija. In: Šercer A, editor. Medicinska enciklopedija. Vol 4. Zagreb: Leksikografski zavod FNRJ; 1960. p. 315-334.

[25] Škarica M. Kako ćemo produžiti život. Zadar: Novinsko poduzeće “Glas Zadra”; 1959.

[26] (1970). Simpozij o gerijatriji.. Lijec Vjesn.

[27] Škarica M. Gerijatrija. In: Popović B, Letica S, Škrbić M, editors. Medicinske struke. Zagreb: Jugoslavenska medicinska naklada; 1981. p. 255-6.

